# Physiological modules for generating discrete and rhythmic movements: action identification by a dynamic recurrent neural network

**DOI:** 10.3389/fncom.2014.00100

**Published:** 2014-09-17

**Authors:** Ana Bengoetxea, Françoise Leurs, Thomas Hoellinger, Ana M. Cebolla, Bernard Dan, Joseph McIntyre, Guy Cheron

**Affiliations:** ^1^Laboratoire de Neurophysiologie et Biomécanique du Mouvement, Faculté des Sciences de la Motricité, Université Libre de BruxellesBrussels, Belgium; ^2^Laboratorio de Cinesiología y Motricidad, Departamento de Fisiología, Facultad de Medicina y Odontología, Universidad del País Vasco-Euskal Herriko Unibertsitatea (UPV/EHU)Leioa, Spain; ^3^Département de Neurologie, Hôpital Universitaire des Enfants Reine Fabiola, Université Libre de BruxellesBrussels, Belgium; ^4^Heath Division, Fondation Tecnalia Research and InnovationSan Sebastian, Spain; ^5^IKERBASQUE – Basque Foundation for ScienceBilbao, Spain; ^6^Laboratoire d'Électrophysiologie, Université de Mons-HainautMons, Belgium

**Keywords:** rhythmic movement, muscular synergy, dynamic recurrent neuronal network, principal component analysis, upper limb, figure-eight

## Abstract

In this study we employed a dynamic recurrent neural network (DRNN) in a novel fashion to reveal characteristics of control modules underlying the generation of muscle activations when drawing figures with the outstretched arm. We asked healthy human subjects to perform four different figure-eight movements in each of two workspaces (frontal plane and sagittal plane). We then trained a DRNN to predict the movement of the wrist from information in the EMG signals from seven different muscles. We trained different instances of the same network on a single movement direction, on all four movement directions in a single movement plane, or on all eight possible movement patterns and looked at the ability of the DRNN to generalize and predict movements for trials that were not included in the training set. Within a single movement plane, a DRNN trained on one movement direction was not able to predict movements of the hand for trials in the other three directions, but a DRNN trained simultaneously on all four movement directions could generalize across movement directions within the same plane. Similarly, the DRNN was able to reproduce the kinematics of the hand for both movement planes, but only if it was trained on examples performed in each one. As we will discuss, these results indicate that there are important dynamical constraints on the mapping of EMG to hand movement that depend on both the time sequence of the movement and on the anatomical constraints of the musculoskeletal system. In a second step, we injected EMG signals constructed from different synergies derived by the PCA in order to identify the mechanical significance of each of these components. From these results, one can surmise that discrete-rhythmic movements may be constructed from three different fundamental modules, one regulating the co-activation of all muscles over the time span of the movement and two others elliciting patterns of reciprocal activation operating in orthogonal directions.

## Introduction

The concept of synergy, associated with basic motor modules of activity, refers to two distinct notions. On the one hand, the large variety of movements accomplished by a limb could be explained by the activation of a reduced number of muscular synergies (Saltiel et al., 2001; Ivanenko et al., 2004; d'Avella et al., 2006). On the other hand, for a given movement, the establishment by the central nervous system (CNS) of synchronous muscular synergies could explain how activity is distributed within a muscle group (Weiss and Flanders, 2004; d'Avella and Bizzi, 2005; Klein Breteler et al., 2007). The first notion gives rise to a simplification in the number of degrees of freedom to be controlled by the CNS for motor control while the second one links modules of activity presented by limb muscles and their functional meaning in the context of motor action. Non-invasive recording of the electromyographic (EMG) signals are widely used to extract muscular synergies (d'Avella et al., 2003, 2008; Ivanenko et al., 2004; Klein Breteler et al., 2007; Cheung et al., 2012; Frère and Hug, 2012). These muscular synergies seem to be structured in the brain stem and spinal cord (Cheung et al., 2009; Clark et al., 2010) and even in the motor cortex for highly skilled movements (Gentner and Classen, 2006; Rathelot and Strick, 2006).

Most attempts to define muscular synergies to date have relied on tools such as principal component analysis or other forms of factor analysis that extract stable relationships (structure) between the activation patterns of multiple muscles. These techniques do not, however, serve to identify structure in the mapping of EMG inputs to the actual motor output (e.g., movement of the hand). In this context, the use of dynamic recurrent neural networks (DRNN) to interpret biological signals coming from the human body could be an interesting complementary approach to extract modules underlying the input-output relationship between muscle activation patterns and movement, where the input signal consist of the EMG signals provided by different muscles implicated in the movement and the output signals of the DRNN would be the movement kinematics. Our proposition is that using a DRNN to map EMGs to kinematics can provide a new, indirect method to better understand motor organization in the CNS, for reasons that we will lay out in following paragraphs.

DRNNs are recognized as universal approximators of dynamical systems (Kuan and Hornik, 1991; Doya, 1996; Yi et al., 2006; Tani et al., 2008; Bicho et al., 2011; Laje and Buonomano, 2013) and the attractor states reached through DRNN learning of EMG-to-kinematic patterns correspond to biologically interpretable solutions (Cheron et al., 1996, 2003, 2006, 2007, 2011; Song and Tong, 2005; Liu and Buonomano, 2009). After the learning phase, the identification performed by the DRNN offers a dynamic memory which has been used, for example, to recognize the physiological preferred direction of action for the studied muscles (Cheron et al., 1996, 2003, 2006, 2007). But the correct recognition by a trained DRNN of EMG patterns not included in the training set may also be related to motor learning, as shown by the following example. When humans learn a specific movement, the initial solutions acquired through self-organized principle are often unstable and become more stable with practice. This feature is apparent in the study by Dominici et al. (2011) where they demonstrated that development of motor patterns from neonatal to toddler consisted of learning new muscle synergies, adding new patterns to the few basic patterns present already at birth. When a DRNN was applied to EMG and kinematic data also acquired from infants and toddlers (Cheron et al., 2001) we showed that it is only when behaviors have been practiced sufficiently by the children and when the task and the context are unchanging that patterns emerged were sufficiently stable to allow the DRNN to generalize (Cheron et al., 2011). Thus, the ability of a DRNN to generalize across movements is a reflection of the stability and maturity of the underlying building blocks. Here we apply a similar concept to analyze a different question, that of how the CNS generalizes the task of programming movements across different kinematic and biomechanical conditions. We hypothesize that the CNS accomplishes this task by exploiting modules to simplify the computation of the motor command. If this hypothesis is valid, then application of the DRNN can be used to characterize which modules are stable across varying situations.

Taking into account Bernstein's theory of motor control (Bongaardt, 2001) where the motor program (also called engram) used to generate a movement is organized at a higher level in the CNS while the details of motor action (also called ecphoria) are selected at a lower level, we can consider that in the EMG command one can find a mixture of the higher (topological) and lower (metrics) aspects of motor action. If we extract the “synchronous synergies” for a given movement, each module could contain different levels of information ranging from the general (what is the form to be reproduced by the hand) to the more specific (what are the joint displacements and muscle activations used to generate the movement of the hand). Applied to the analysis of a drawing movement, such as a figure-eight, we should find in the EMG signals information corresponding to a generalized “figure-eight” motor program mixed with the information corresponding to the specific aspects of motor execution, such as the movement's velocity, amplitude, joint configuration and biomechanical constraints.

We chose to study figure-eight movements. These gestures require the displacement of the end-effector segment through all the directions within the plane of the figure. Note, however, that starting from the central point one can perform this figure with one of four different initial directions. Given the fact that EMG patterns are modulated by movement direction in 3D space (Flanders et al., 1994, 1996; Hoffman and Strick, 1999), forcing the DRNN to converge to any one of these four patterns of movement should create an attractor state that reflects the directional tuning of synergies within the workspace. Given also that a muscle's activation depends on its mechanical action, which in turn depends on joint configuration (Hogan, 1985; Buneo et al., 1997), a DRNN that converges to the four figure eights realized in one part of the joint workspace may or may not recognize muscle activities when the same movements are performed in a different workspace region. Finally, considering that the precise structures of some muscle synergies are subject-specific (Torres-Oviedo and Ting, 2010), a DRNN trained with all the figure-eight movements of one subject may or may not detect the tuning synergies of another, depending on how stable the underlying modules are across subjects. We therefore set out to measure the ability of a DRNN to learn and recognize movements from EMG signals for figure-eight movements performed in different directions and in different parts of the workspace as a mean to assess the invariance of movement modules or primitives across a variety of movement conditions. We also used the DRNN in a novel fashion to identify the physical manifestation, in terms of hand kinematics, of synchronous synergies (d'Avella and Bizzi, 2005; Klein Breteler et al., 2007) identified by principal component analysis in our previous study (see companion paper, this issue).

## Material and methods

Data were collected from five right-handed subjects aged between 21 and 40 years. All were in good health, free from known neurological disorders, and had given informed consent to take part in the study, which was approved by the local ethics committee. They were asked to draw, as fast as possible, two series of figure-eight movements in free space with the right arm fully extended at the elbow (for more details see Bengoetxea et al., 2010). Movements were initiated in the center of the figure with an initial up-right (UR), down-right (DR), up-left (UL) or down-left (DL) direction with respect to external coordinates. Three subjects performed the task in both the frontal and sagittal workspaces (in separate sets of trials) depending on the flexion or abduction posture of the shoulder. Two additional subjects (subjects 4 and 5) performed the movements only in the frontal workspace.

### Data acquisition

Movements of the index finger were recorded and analyzed using the optoelectronic ELITE system (2 CCD-cameras, sampling rate of 100 Hz; BTS, Milan; Ferrigno and Pedotti, 1985). The cameras were placed 4 m apart from each other and 4 m from the subject. Four markers were attached to the arm (on the acromion, the lateral condoyle of the humerus, the radial apophysis of the wrist and the index finger). Velocity signals were obtained by digitally differentiating position signals using a fifth-order polynomial approximation. Reconstruction of the arm movements by the ELITE system using the trajectories of the 4 markers confirmed the visual observation that the upper arm, forearm, hand and index finger acted as a rigid link (Bengoetxea et al., 2010). Thus, we analyzed here only the marker on the index finger that was used to trace the figure-eight.

Surface electromyographic activity (EMG) was recorded with the TELEMG system (BTS, Milan) synchronized with the kinematic data. Silver-silver chloride electrode pairs (inter-electrode distance of 2.5 cm) were placed over the belly of the following 7 muscles: posterior deltoid (PD), anterior deltoid (AD), median deltoid (MD), pectoralis major superior and inferior (PMS and PMI), latissimus dorsi (LD), and teres major (TM). Raw EMG signals (differential detection) were amplified by a portable unit with a gain of 1000 and transmitted to the main unit via a telemetry system (Telemg, BTS). A functional resistance test that isolated specific muscles was made in order to verify the absence of cross talk between adjacent muscles. Thereafter, EMGs were band-pass filtered (10–500 Hz), digitized at 1 kHz, full-wave rectified and smoothed by means of a third-order averaging filter with a time constant of 20 ms (Hof and Van den Berg, 1981).

### Dynamic recurrent neural network

We used a DRNN, that consisted of 50 fully connected hidden neurons, 7 input neurons and 2 output neurons. The network included a looping mechanism (fully connected structure) that enables this network to learn and store information (memory). This feature allows the network to model complex situations with multiple influences. This particular DRNN structure has varying time constants as well as varying weights for the artificial neurons. The adaptive time constants make the DRNN dynamic and therefore capable of modeling time varying input and outputs.

The DRNN was governed by the following equation:

(1) $$T_{i}\;dy_{i}/dt=-y_{i}+F\left(x_{i}\right)+I_{i}$$

where *F*(*α*) is the squashing function

$$F\left(\alpha \right)={\left(1+e^{-\alpha }\right)}^{-1},$$

*Y_i_* is the state or activation level of unit i, *I_i_* is an external input (or bias), and *x_i_* is given by

(2) $$x_{i}=\sum_{j}w_{ij}\;y_{j},$$

which is the propagation equation of the network (*x_i_* is called the total or effective input of the neuron, and *w_ij_* is the synaptic weight between units *i* and *j*). The time constant *T_i_* acts like a relaxation process, allowing a more complex dynamical behavior and improving the non-linearity effect of the sigmoid function (Cheron et al., 1996; Draye et al., 1996, 1997). In order to make the temporal behavior of the network explicit, an error function is defined as

(3) $$E=\int_{t_{0}}^{t_{1}}q\left(y\left(t\right),t\right)dt$$

where *t*_0_ and *t*_1_ give the time interval during which the correction process occurs. The function *q*(*y*(*t*), *t*) is the cost function at time *t* which depends on the vector of the neuron activations *y* and on time *t*. We then introduce new variables *p_i_* (called adjoint variables) that are determined by the following system of differential equations:

(4) $$\frac{dp_{i}}{dt}=\frac{1}{T_{i}}\int_{t_{0}}^{t_{1}}p_{i}-e_{i}-\sum_{j}\frac{1}{T_{j}}w_{ij}F^{\prime }\left(x_{j}\right)p_{j}$$

with boundary conditions *p*_i_(*t*_1_) = 0. After the introduction of these new variables, we can derive the learning equations:

(5) $$\frac{\partial E}{\partial w_{ij}}=\frac{1}{T_{i}}\int_{t_{0}}^{t_{1}}y_{i}F^{\prime \left(x_{j}\right)p_{j}}dt;\frac{\partial E}{\partial T_{i}}=\frac{1}{T_{i}}\int_{t_{0}}^{t_{1}}p_{i}\frac{dy_{i}}{dt}dt$$

The training of the DRNN was supervised, involving learning-rule adaptations of the synaptic weights and time constants of each unit (for more details, see Draye et al., 1997). This algorithm, called “backpropagation through time,” aims to minimize the error value defined as the differential area between the experimental and simulated output kinematics signals.

#### DRNN learning strategy

**Figure 1:**
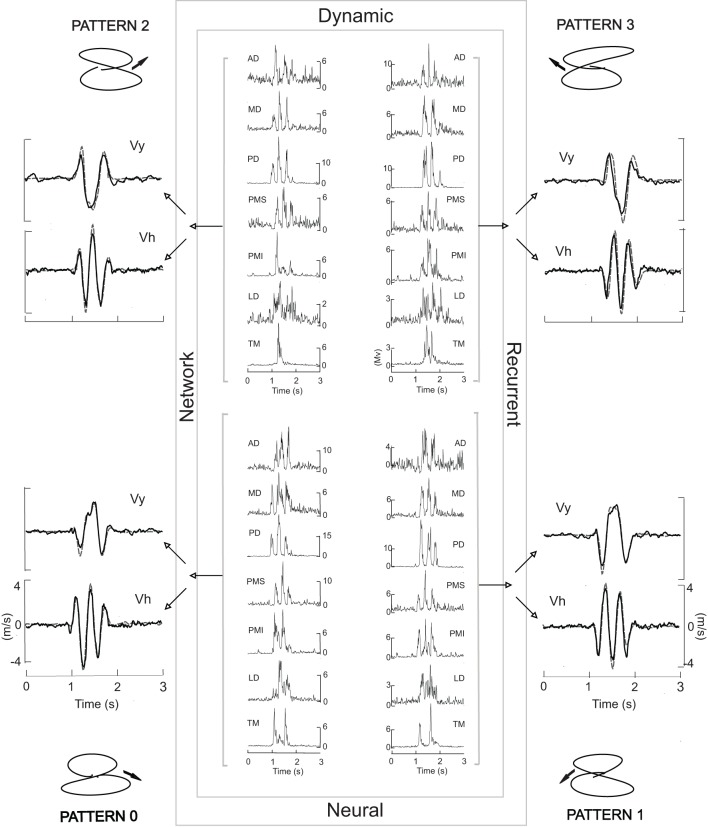
**The central box symbolizes the DRNN**. Inside each corner of the box the four EMG patterns used to train the network are illustrated, one for each of the four movement patterns shown outside the box. Each EMG signal from a given movement was sent to all 50 artificial neurons (hidden units) which converge on two output units acting merely as summators. One output neuron provides the vertical component of the finger velocity, the other the horizontal component. Each iteration of the training was performed with one pattern at a time in random order. Black velocity profiles represent the learned output. Gray dashed velocity profiles correspond to the experimental data. The corresponding experimental trajectory is illustrated in each corner of the figure.

The DRNN used here was adapted from a previous version originally developed for the reproduction of a figure eight (Cheron et al., 1996; Draye et al., 1997). Although in our previous study we showed that the DRNN could recognize the preferential direction of the muscles based on a single movement, it was not able to generalize from training on one movement to reproduce movements based on EMG signals from trials with different initial directions of the movement. In order to obtain this ability to generalize, we developed a new learning procedure called “multi-pattern learning” (Figure 1). In this figure we illustrated a multi-pattern learning for four movements realized in the frontal workspace, each one corresponding to a figure eight initiated in one different direction. This DRNN was alternatively trained in sequential iterations on one of the four patterns, in a random sequence. Three types of multi-pattern training were performed, the first one with the 4 movements realized in the frontal plane, the second one with the 4 figure-eight movements realized in the sagittal plane and the third one with the 8 figure-eight movements taken from both planes. We compared the results of these training processes to the results obtained when trained on a single movement, as reported in our previous publication.

#### DRNN generalization

After training on data from a given workspace, EMG profiles corresponding to novel movements from either the same workspace or from the other workspace were fed into the trained DRNN. A comparison was then made between the velocity profiles predicted by the DRNN and the actual measured movements of the index finger. In order to quantify the resemblance between the measured and simulated velocity profiles, we calculated a similarity index (SI), using the following equation:

(6) $$SI=\frac{\int f_{1}\left(t\right)f_{2}\left(t\right)dt}{{\left[\left(\int f_{1}{\left(t\right)}^{2}dt\right)\left(\int f_{2}{\left(t\right)}^{2}dt\right)\right]}^{\frac{1}{2}}}$$

We looked for the effect of the injection of novel EMG profiles in each of the 3 types of multi-pattern DRNN. For statistical analysis, we first tested for normality in the distributions of SIs, using the Kolgomorov–Smirnov test. We then used a repeated-measures ANOVA followed by Scheffe's test for *post-hoc* analyses (Statistica ©Statsoft).

#### Synergy action identification via DRNN simulation

In the second part of our study we explored the physiological meaning of purported muscle synergies by reconstructing the EMG signals based on different combinations of components computed by principal component analysis and injecting them into the DRNN. The methods used to compute the principal components as well as an analysis of the resulting synergies are presented in our companion paper published in this issue. We limited this analysis to the first three PCs, which accounted for at least 75.28% of the total variance in each movement (mean across movements: 83.01 ± 2.84% of the total variance). For each muscle we reconstructed the EMG signals from components PC1, PC2, and PC3 individually and compound EMG signals constructed from PC1&2, PC1&3, PC2&3, and PC1&2&3, plus the movements predicted from the full EMG signal (i.e., PC1-7), for a total of 8 different sets of EMG signals. These sets of EMG signals were then injected into the DRNN that has been trained on figure eight movements performed in all four directions in both the frontal and sagittal planes. A comparison was then made between the velocity profiles predicted by the DRNN and the actual measured movements of the index finger, using the SI. We performed a similar procedure for EMG signals reconstructed from the first three factors after a varimax rotation.

## Results

We first looked at the ability of the DRNN to predict patterns of movement from EMG signals as a function of the set of movements used to train the network. Figure 1 illustrates the typical performance of the DRNN trained on the EMG patterns (center) and movement recordings from a set of 4 frontal workspace movements. To each side of the rectangle, representing the DRNN, we have superimposed the learned (black curves) and the measured (gray dashed curves) velocity profiles. After a learning phase involving 15,000 iterations, the DRNN trained on this set of movements in the frontal workspace was able to reproduce the horizontal and vertical velocity profiles of the training set with a mean error value of 0.004 ± 0.001.

**Figure 2:**
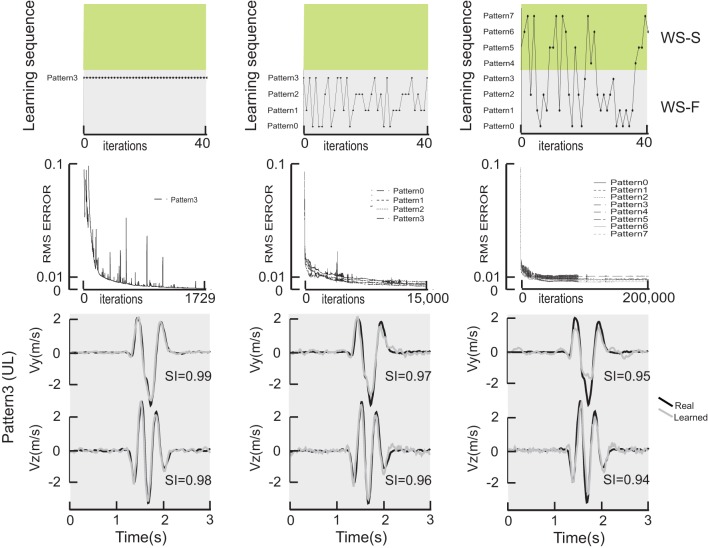
**Comparison of learning performance of the network for different learning strategies**. The top row illustrates the sequence for a 1-pattern learning (left column), for a 4-pattern learning (middle column) and finally an 8-pattern learning (right column). Green and gray surfaces represent the frontal and sagittal workspaces. The first 40 iterations for each training session are plotted in the top row showing the sequence of movements presented to the network on each iteration. The middle row shows the RMS error as a function of iteration in the learning procedure. The bottom row shows the actual, measured hand velocities (Vy and Vz) for a single movement pattern (pattern #3, which started in the upward/leftward direction) compared to the simulated hand velocity produced by each of 3 DRNNs, after 1-pattern (left), 4-pattern (middle) and 8-pattern (right) learning.

Figure 2 shows a comparison of learning performance of the network for different learning strategies. The learning sequence for 1-pattern learning (left column), for 4-pattern learning corresponding to the figure-eight movements realized in the frontal workspace (middle column) and 8-pattern learning trained with the movements realized in the frontal and sagittal plane and with the 4 different initial directions (right column). The first 40 iterations for each training session are plotted in the top row showing the sequence of movements presented to the network on each iteration, thus illustrating the difference between the three training strategies.

The middle row of Figure 2 shows the RMS error as a function of iteration in the learning procedure. Note the change in scale on the X axis. One can observe that the learning error only reached the value of 0.001 that we observed in our previous studies for the single pattern training, despite the greater number of iterations performed for the multi-pattern trainings. For the multi-pattern training illustrated here, the mean error was 0.004 ± 0.001 and 0.009 ± 0.002 for 4- and 8-pattern training, respectively. In terms of the ability of the network to converge to a stable response, essentially all networks starting from a random set of initial synaptic weights and time constants and trained on a single movement pattern converged to a stable response. In contrast, out of the 90 multi-pattern trainings with 4 movements that were initiated, 32.5% had asymptotic error curves with a mean error of 0.016 ± 0.21. Similarly, out of the 79 sessions initiated for multi-pattern trainings with 8 movements, 21.5% had asymptotic error curves with a mean error of 0.011 ± 0.002.

The bottom row of Figure 2 shows the actual, measured hand velocities (Vy and Vz) for a single movement pattern (Pattern #3, which started in the upward/leftward direction) compared to the simulated hand velocity produced by each of 3 DRNNs, after 1-pattern (left), 4-pattern (middle) and 8-pattern (right) learning. It is interesting to note that for the three learning conditions shown in Figure 2, the temporal relationship was well reproduced in all three cases between the real and the learned velocity profiles. The only differences between the actual and reconstructed hand trajectories appeared in the magnitude of the peak velocities, as can be seen in the example shown here. These qualitative differences were reflected in the similarity index for each of the velocity components for this particular movement. For the example shown, one can see that the SI for the vertical component decreased from 0.99 to 0.97 to 0.95, and for the horizontal component from 0.98 to 0.96 to 0.94, for 1-, 4-, and 8-pattern training, respectively.

**Figure 3:**
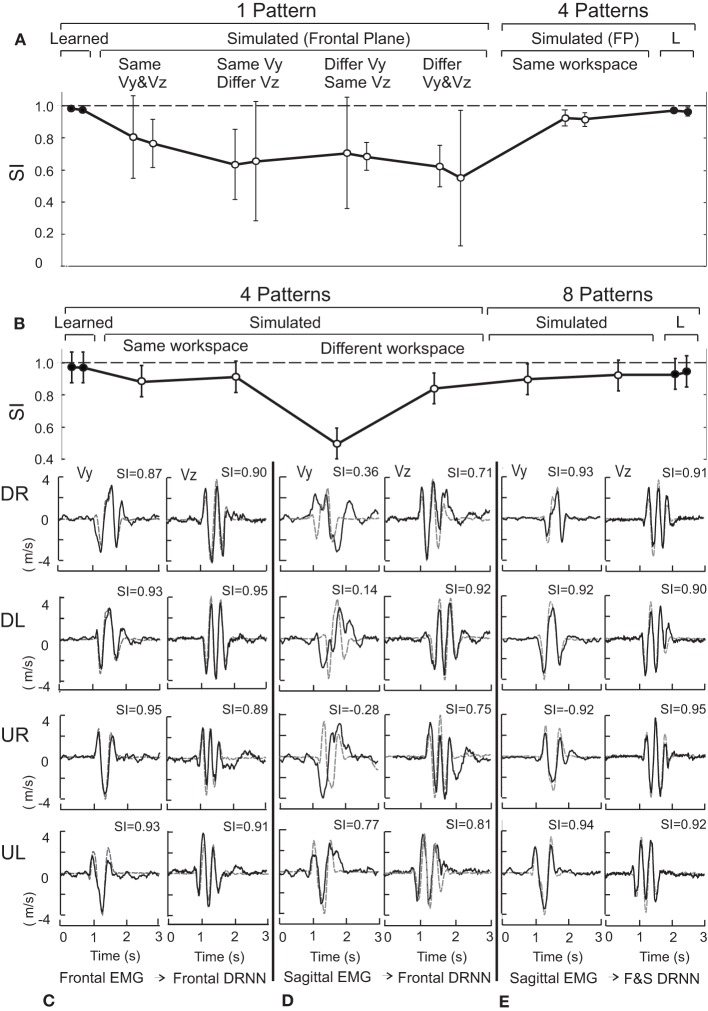
**(A)** Shows the mean and standard deviation of the similarity index for the same and different movement directions within the frontal plane for 1-pattern DRNN and 4-pattern DRNN trainings. **(B)** Presents similarity indexes for same and crossed workspaces for 4-pattern DRNN and 8-pattern DRNN trainings. SIs for learned and simulated curves correspond to black and open circles, respectively. **(C)** Illustrates the simulated and experimental velocity profiles for the four frontal movements (DR: down-right; DL: down-left; UR: up-right; UL: up-left) injected into the frontal DRNN. **(D)** Shows the simulated velocity profiles from injecting the sagittal EMG patterns into the frontal DRNN. **(E)** Shows the simulated velocity profiles from injecting the sagittal EMG patterns into the dual DRNN. The similarity indexes are indicated above each profile's graph.

The mean SI ± SD for the vertical and the horizontal velocity components were respectively 0.98 ± 0.004 and 0.97 ± 0.001 for the 1-pattern training, 0.97 ± 0.01 and 0.97 ± 0.02 for the 4-pattern training and 0.93 ± 0.02 and 0.95 ± 0.01 for the 8-pattern training. These values are illustrated in Figure 3A for the 1-pattern and 4-pattern training and in Figure 3B for the 4-pattern and 8-pattern learning (filled circles). A Kolgomorov–Smirnov test showed that the SIs followed normal distribution for the two velocity components and for the three types of training. To test the ability of each training method to reproduce the movements within the respective training sets, we performed repeated-measures ANOVA on the SIs with training type (1-pattern, 4-pattern, 8-pattern) and velocity component (Vy, Vz) as independent factors. The ANOVA showed a significant main effect of training type [*F*_(2, 14)_ = 36.92; *p* < 0.001] and Scheffe's *post-hoc* analysis showed that the DRNN trained on 8-patterns reproduced significantly less accurately the velocity curves than either the 4- and 1-pattern trained DRNNs (*p* < 0.001 for both comparisons). There was no main effect of velocity component and although there was a significant cross effect between the two factors [*F*_(2, 14)_ = 6.04, *p* = 0.0128], indicating a difference in the way that the SIs changed for the two velocity components across training types, *post-hoc* analysis did not detect a significant difference between SIs for Vy and Vz within any of the three training types.

### DRNN generalization

To test the ability of each DRNN to predict movement patterns from EMG signals that were not in the training set, we injected various EMG patterns, corresponding to figure-eights initiated with different directions and realized in both workspaces, as inputs to the DRNN and compared the output of the network to the corresponding movement. For the generalization phase we used only the DRNN instances with the best mean error for each learning condition. This set of 7 trained DRNNs (4 1-pattern, 2 4-pattern, and 1 8-pattern) was used to test the ability of the networks trained in each fashion to generalize across movements within the same movement plane, to generalize across movement planes and to generalize across subjects, as we will describe in the following paragraphs.

#### Generalization between movement directions

Consider first the generalization across movement patterns within the same workspace. Four instances of the DRNN were each exposed during the training phase to a single movement and the corresponding EMG recordings in the frontal plane, one for each of the four possible directions of movement. We then injected EMG patterns from four additional movements performed by the same subject in the frontal plane into each of the four instances of the DRNN (for a total of 16 EMG/DRNN pairings) and computed the similarity index between the predicted and actual movement velocities in Y and Z. The resulting similarity indices were then divided into four groups according to the pairing between the test movement and the movement on which the particular instance of the DRNN was trained. Four pairings consisted of test and training movements that started in the same direction in both Y and Z. Four pairings consisted of test and training movements that started in the same direction in Y but opposite directions in Z while conversely, four pairings consisted of test and training movements that started in the same direction in Z but opposite directions in Y. Finally, four pairings consisted of training and test movements that started in opposite directions in both Y and Z. To this we added one additional pairing in which each of the four test movements were injected into an instance of the DRNN that had been exposed to all four movements from the training set according to the multi-pattern learning scheme depicted in Figures 1, 2.

Although an instance of the DRNN trained on a single movement converged to a very low RMS error for predicting the velocity of the hand from the EMG used to train the DRNN, such 1-pattern trained DRNNs did a poor job, in general, of reproducing from EMG signals figure-eight movements that were not included in the training set (mean SI for the vertical and horizontal velocity components were 0.63 ± 0.22 and 0.68 ± 0.15, respectively, across movements in all four directions). Of greater interest is the effect of movement direction on the ability of a single-pattern DRNN to reproduce the movement. When the EMG came from a movement not in the training set, but initiated in the same direction as the training movement in both Y and Z, the mean values of SI were 0.80 ± 0.16 and 0.76 ± 0.09 for the vertical and horizontal velocity components, respectively. When the test and training movements shared the same initial vertical component but had opposite initial horizontal component, the mean SIs were 0.63 ± 0.14 and 0.66 ± 0.23 for Vy and Vz, respectively, while when the two movements shared the same initial horizontal component but opposite vertical component, the corresponding SIs were 0.71 ± 0.22 and 0.68 ± 0.05. SIs were lowest when both vertical and horizontal component were different, with values of 0.62 ± 0.08 and 0.55 ± 0.27, respectively. An ANOVA with factors *velocity component* and DRNN/direction *pairing* showed a significant effect of the pairing factor [*F*_(4, 12)_ = 4.8398, *p* = 0.01477), with no significant difference between Vy and Vz and no cross effect. The DRNN trained on all 4 patterns from the same workspace was much better able to reproduce the kinematics generated from the EMG recordings for other movements performed within that workspace. Indeed, the 4-pattern DRNN, with average SIs of 0.92 ± 0.03 and 0.91 ± 0.03 for Vy and Vz, respectively across all movement directions (Figure 3A), was better able to reproduce the hand velocities than a 1-pattern trained DRNN could for figure eights performed in the same direction as it's own training movement.

#### Generalization between planes

Next we considered the ability of a DRNN to generalize between different parts of the workspace (i.e., different movement planes). For the frontal and sagittal movements, each simulated by the appropriate frontal and sagittal DRNNs, the mean SIs for the vertical and horizontal velocity components were 0.89 ± 0.06 and 0.91 ± 0.03, respectively (Figure 3B, “same workspace”). Figure 3C illustrates a typical pair of simulated velocity profiles (Vy and Vz) computed from EMG signals taken during movements in the frontal plane that were not in the training set, overlaid on the actual velocity profile.

DRNNs trained on all four movement patterns within one plane were nevertheless much less able to predict the hand trajectories from EMG signals recorded from movements in the other. Figure 3D illustrates the simulated and actual velocity profiles for the injection of the EMG patterns from a sagittal movement into the DRNN trained on the 4 frontal movements. While the predictions of the horizontal velocity profiles (Vz) achieved levels of SI similar to that produced by the 4-pattern DRNN for the same workspace, the DRNN did not successfully reproduce the vertical velocity component (Vy) across workspaces. The mean SI for simulated movements computed by the 4-pattern DRNN trained on the “other” workspace was 0.84 ± 0.06 for the horizontal component and 0.50 ± 0.13 for the vertical component (Figure 3B, “different workspace”). On the other hand, an 8-pattern DRNN trained on movements from both workspaces was able to reproduce movements in either workspace just as well as each of the 4-pattern DRNNs were able to reproduce movements within their own workspaces. For the 8-pattern DRNN, the mean similarity index for the vertical and horizontal velocity components was 0.90 ± 0.07 and 0.92 ± 0.02, respectively (Figure 3B, “8 patterns”).

We used ANOVA to test for statistical significance of the observations described above. From all the movements recorded for the one subject whose data was used to train the networks, we injected the EMG signals from all the other movements that were not in the training sets into each of three instances of the DRNN, the one that had been trained on 4 movements in the frontal plane, the one that had been trained on 4 movements in the sagittal plane and one that had been trained on all 8 movements, resulting in a total of 8 × 3 = 24 simulated movements. We then used the pairing between the DRNN and the actual movement's workspace to divide the 24 simulated movements into 3 groups of 8 movements each, those produced by the 4-pattern DRNN trained on movements from the same plane, those produced by the 4-pattern DRNN trained on the other plane and those produced by the 8-pattern DRNN trained on both planes. This resulted in a 3 × 2 multifactor ANOVA, with *DRNN/EMG pairing* (same-plane, cross-plane, and dual-plane) and *velocity component* (Vy, Vz) as within-group factors (i.e., a repeated measure for the same movement produced by the subject). Note that the normality of our data set was first verified by the Kolgomorov–Smirnov test before the ANOVA was applied.

The ANOVA described above revealed a highly significant main effect for the type of DRNN/EMG pairing [*F*_(2, 14)_ = 23.61; *p* = 0.0003]. There was a significant main effect of velocity component [*F*_(1, 7)_ = 7.58, *p* = 0.0284] and a significant interaction [*F*_(2, 14)_ = 6.04, *p* = 0.0129]. Scheffe's *post-hoc* analysis showed that there was no significant difference between the ability of each of the three DRNN/EMG pairings to reproduce the horizontal velocity component (Vz). On the other hand, the SIs for the vertical velocity component (Vy) were significantly lower (worse) for the cross-plane simulations than for the simulations produced by either the same-plane or the dual-plane DRNN/EMG pairings (illustrated in Figure 3B).

**Figure 4:**
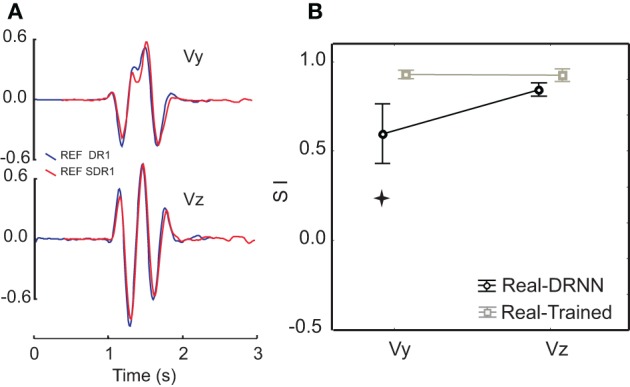
**Comparison of similarity between actual and predicted movements between the two planes. (A)** An example of velocity profiles for a single movement pattern performed in the frontal (blue) and sagittal (red) planes. **(B)** Similarity indexes for the comparison of actual movements performed in the frontal and sagittal planes, overlaid with the similarity indexes for movements reconstructed from EMG collected in one plane by a DRNN trained on movements performed in the other plane. The star shows SIs that were significantly different (*p* < 0.005).

As a control, we considered whether the DRNN's inability to predict movements across planes could be attributed to differences in the kinematics of the figure-eight movements performed in each plane. Figure 4A shows a comparison of the velocity components for pattern #3 for the reference subject, performed in the frontal (blue) and sagittal (red) planes. One can observe that the movements were very similar both in terms of velocity amplitude and in terms of the temporal characteristics. Figure 4B shows a comparison of the mean similarity index (SI) computed between the real test movement in one plane and the corresponding training movement from the other plane (real-trained) and between the real test movement from one plane and the movement predicted from the corresponding EMGs by the DRNN that had been trained on movements from the other plane. One can see that on average the SIs between actual movements in different planes were high for both the vertical and horizontal velocity components (0.93 ± 0.04 and 0.92 ± 0.06, respectively). SIs between actual movements and movements predicted by the DRNN from EMGs were somewhat lower, especially for Vy (0.93 ± 0.04 and 0.6 ± 0.3). Statistical analyses revealed that although there was no difference between the SI for the comparison of real movements and predicted movements for the horizontal velocity component (Vz), there was a significantly lower similarity between predicted and actual movements for the vertical component (Vy) (Scheffe's *post-hoc*: *p* < 0.005), compared to the similarity of actual movements performed in different planes [*F*_(1, 14)_ = 10.986, *p* = 0.00511]. In other words, the inability of the DRNN to generalize across movement planes in terms of Vy cannot be attributed to differences in the movement kinematics performed in each plane.

**Figure 5:**
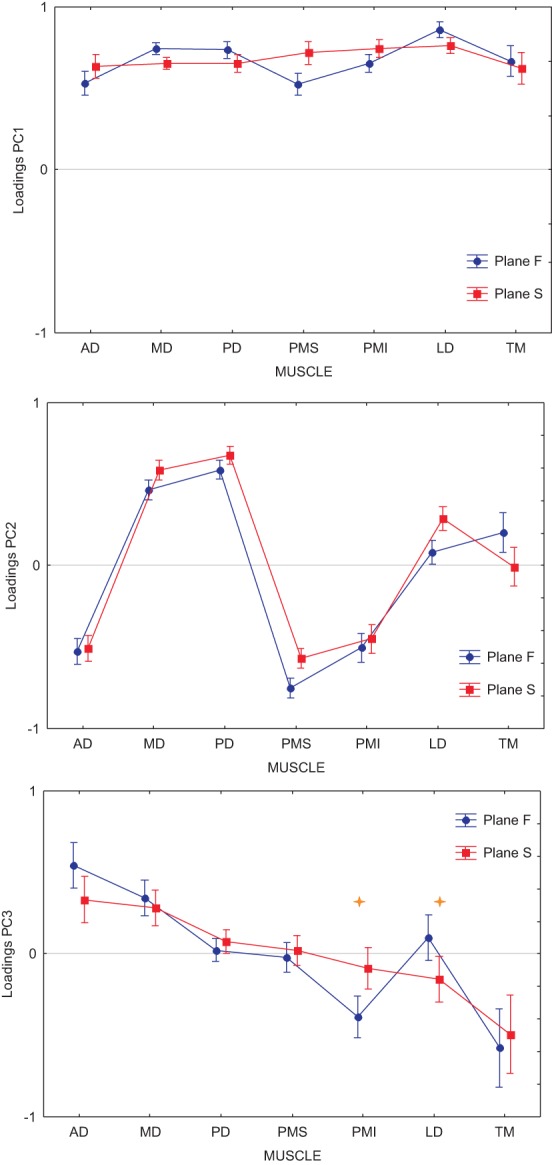
**Average loadings for each PC and each muscle, computed for each of the two movement planes**. Each graph shows the loading for Anterior Deltoid (AD), Medial Deltoid (MD), Posterior Deltoid (PD), Pectoralis Major Superior (PMS), Pectoralis Major Inferior (PMI), Latissimus Dorsi (LD), and Teres Major (TM). In blue are represented the loadings for movements performed in the frontal workspace, in red the loadings for movements performed in the sagittal workspace. Stars show loading for individual muscles that were significantly different between the frontal and sagittal planes (*p* < 0.001).

Finally, we asked whether the inability of the DRNN to generalize across movement planes could be related to changes in muscle synergies as identified through principal component analysis of these same movements and EMG. In our companion paper we showed that the loading vectors (synergies) varied, on average across subjects, between figure eights drawn in the frontal and sagittal planes. Figure 5 shows the average loading for each PC, computed for each of the two movement planes, for the reference subject alone. We performed an ANOVA on the loadings with movement plane (frontal or sagittal) as a grouping factor and muscle (AD, MD, PD, PMS, PMI, LD, TM) as a repeated measure. For each of the 3 PCs, there was a significant main effect of the muscle factor, a significant main effect of movement plane and a significant cross effect between the two. But this ANOVA was not performed with these global contrasts in mind. The pertinent test from this analysis was the *post-hoc* analysis that we applied to determine which muscle loadings, if any, changed between the two movement planes within each PC. For PC1 and PC2, Scheffe's *post-hoc* test detected no significant changes in individual muscle loadings between the frontal and sagittal planes. For PC3, however, there was a significant increase in the loading of LD and a significant decrease in the loading of PMI when passing from the frontal to the sagittal movement plane.

#### Synergy action identification via DRNN simulation

**Figure 6:**
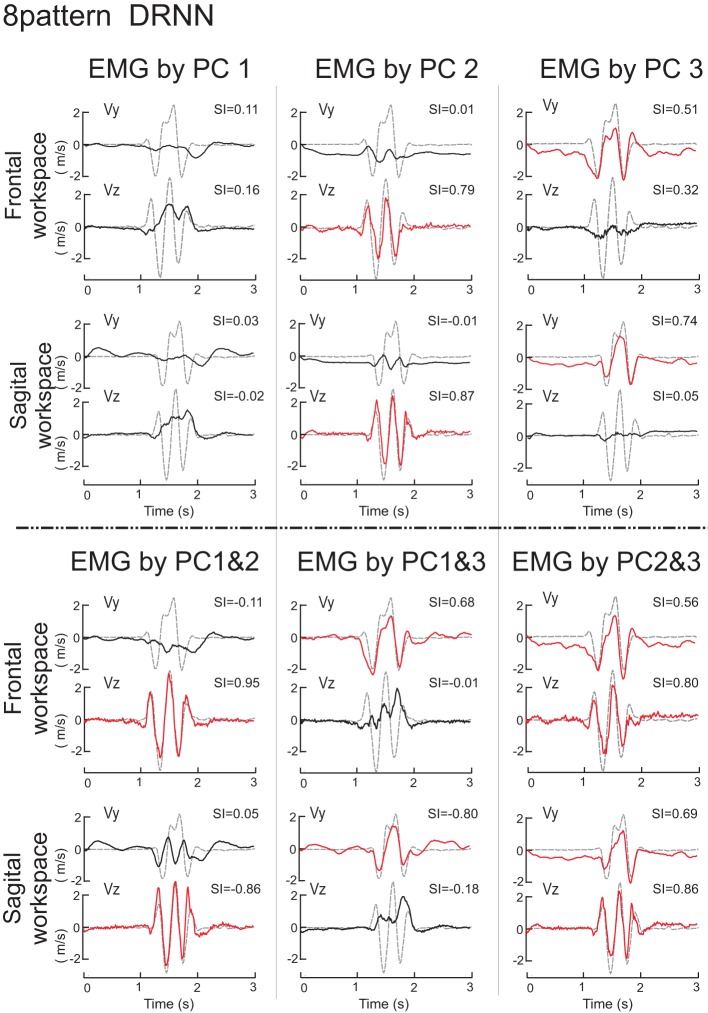
**Simulations with the 8-pattern DRNN trained on move- ments from both planes**. In this figure are illustrated the simulations for EMG signals reconstructed by means of PC1, PC2, PC3 (top part of the figure) and PC1&2, PC1&3, and PC2&3 (bottom part) for the same figure-eight movement (initiated down and to the right) realized in the frontal (first row) and sagittal (second row) workspaces. Real curves are represented by gray dashed traces and simulated curves by black and red traces. Traces were drawn in red when the SIs exceeded a threshold arbitrarily set to 0.5. SIs are indicated in the upper right corner of each graph.

**Figure 7:**
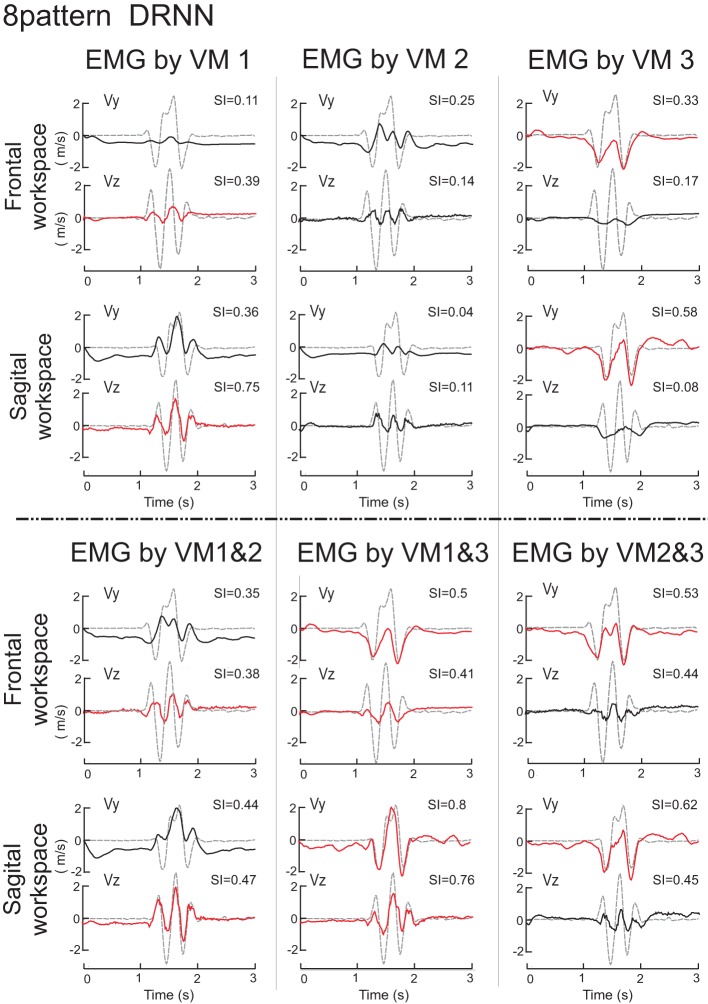
**Simulations with the 8-pattern DRNN trained on movements from both planes**. In this figure are illustrated the simulations for EMG signals reconstructed by means of VM1, VM2, VM3 (top part of the figure) and VM1&2, VM1&3, and VM2&3 (bottom part) for the same figure-eight movement (initiated down and to the right) realized in the frontal (first row) and sagittal (second row) workspaces. Real curves are represented by gray dashed and simulated curves by black and red lines. SIs are indicated in the upper right corner of each graph.

We then set out to see how the DRNN would interpret the action of EMG signals associated with each of the different components identified by principal component analysis and by varimax factor analysis (see companion paper). The simulation phase consisted of sending to the 8-pattern (dual plane) trained DRNN the EMG signal reconstructed with the first, second and third components and the combinations of components 1&2, 1&3, 2&3, and 1&2&3 for both the principal component (Figure 6) and varimax (Figure 7) decompositions. In these figures are illustrated the simulations for EMG signals taken from the reference subject for the same figure-eight movement (initiated down and to the right) realized in the frontal and sagittal workspaces.

#### Principal components

The superposition of real (gray dashed curves) and simulated (black and red curves) velocity curves in Figure 6 shows that the EMG signals composed by PC2 and PC3 alone reproduced very nearly the horizontal and vertical velocity component, respectively, while the EMG signal reconstructed from PC1 alone produced simulated movements that did not resemble either of the velocity components. Nevertheless, combining PC1 with either PC2 or PC3 (lower part of Figure 6) increased the level of reproduction for the horizontal and vertical component respectively, compared to any one component alone. Indeed, PC1 seems to have an influence for the stability in the static phase existing before and after the movement, as we can see in the simulation velocity curves resulting from EMG reconstructed with the second and third, but not the first PCs combined. Compared to the simulation from PC1, PC1&2, and PC1&3, the simulated movements that did not contain PC1 (PC2, PC3, PC2&3) exhibited a negative bias in Vy both before and after the figure-eight movement, indicating that without the PC1 component the hand would drift downwards.

We used ANOVA to test statistically the ability of each PC or combination of PCs to reproduce the velocity profiles of the actual movements. We compared the similarity indexes between the 8 measured movement patterns for the reference subject with the simulated movements from the eight different DRNN reconstructions from the corresponding EMG signals (PC1, PC2, PC3, PC1&2, PC1&3, PC2&3, PC1&2&3, PC1-7 = real EMG). Recall that the EMG signals and movement recordings that were used to simulate movements via the DRNN were different from the EMG and movement recordings used to the train the DRNN. This resulted in an ANOVA with two repeated-measures factors (PC combination, velocity component). The repeated measures ANOVA showed that the SIs were significantly different depending on which combination of PCs were used to reconstruct the EMG signal [main effect: *F*_(7, 49)_ = 144.36, *p* < 0.0001]. Sheffe's *post-hoc* analysis showed that SIs obtained with EMG reconstructed from P1&2&3 were not significantly different from the movements simulated with the real EMG (mean SI: 0.80 ± 0.02 and 0.91 ± 0.01, respectively; *p* > 0.99]. SIs for movements simulated with PC2&3 (0.78 ± 0.02) were significantly lower than those simulated from the real EMG (*p* = 0.046), but only slightly worse than those simulated with PC1&2&3 (*p* > 0.99). For all the other simulated movements with PC1, PC2, PC3, PC1&2, PC1&3, or PC2&3 (mean SI 0.05 ± 0.04, 0.42 ± 0.03, 0.37 ± 0.02, 0.45 ± 0.03, 0.36 ± 0.03, respectively), the SIs were significantly lower than the reconstruction from the full EMG signal (*p* < 0.001).

The repeated measures ANOVA did not show a significant main effect of the velocity component factor [*F*_(1, 7)_ = 4.26, *p* = 0.078] but there was a highly significant interaction between the velocity component and the PC combination factors [*F*_(7, 49)_ = 117.95, *p* < 0.0001]. Scheffe's *post-hoc* analysis confirmed the results illustrated in Figure 6:

1. Simulation of the movements based on the EMG signals in PC2 reproduced the horizontal velocity component of figure-eight movements but not the vertical one (*p* < 0.001). The mean SIs for the horizontal and vertical velocity component were 0.82 ± 0.03 for Vz and 0.03 ± 0.05 for Vy.
2. The simulated movements corresponding to PC3 reproduced the vertical velocity component but not the horizontal one (*p* < 0.001). The mean SI for the horizontal and vertical velocity component were −0.02 ± 0.04 for Vz and 0.76 ± 0.03 for Vy.
3. The EMG reconstructed with PC1&2, and with PC1&3 confirmed the preceding results. The mean SI for the horizontal and vertical velocity component were 0.89 ± 0.02 and 0.01 ± 0.06 for PC1&2, and 0.02 ± 0.06 and 0.70 ± 0.04 for PC1&3, respectively.

There were no differences in SIs for the horizontal and vertical velocity components for EMGs composed with PC2&3, with PC1&2&3 or for the real EMG (*p* > 0.99). The mean SI for the horizontal and vertical velocity component were 0.81 ± 0.03, and 0.74 ± 0.02, 0.88 ± 0.02 and 0.72 ± 0.03, 0.92 ± 0.01 and 0.90 ± 0.02, respectively.

#### Varimax

The simulation of the velocity traces by the DRNN based on EMG signals reconstructed from the varimax decomposition (Figure 7) were more difficult to interpret in terms of the actions of each component. We display the velocity signals in this figure in black or red depending on whether the SIs for the varimax-reconstructed EMGs were significantly different from the best scores obtained for the principal component (Figure 6). From this one can see that no single varimax component produced very well either of the velocity components. Even the traces shown in red manifest noticeable differences between the actual and predicted velocities. While VM3 produced the same number of peaks in Vy, the predicted velocities were strictly negative while the real velocity had both positive and negative components. For VM1, the predicted and actual Vz showed similarities in the number of peaks, but the DRNN predicted velocities that were not nearly as strong as the actual measured velocities.

**Figure 8:**
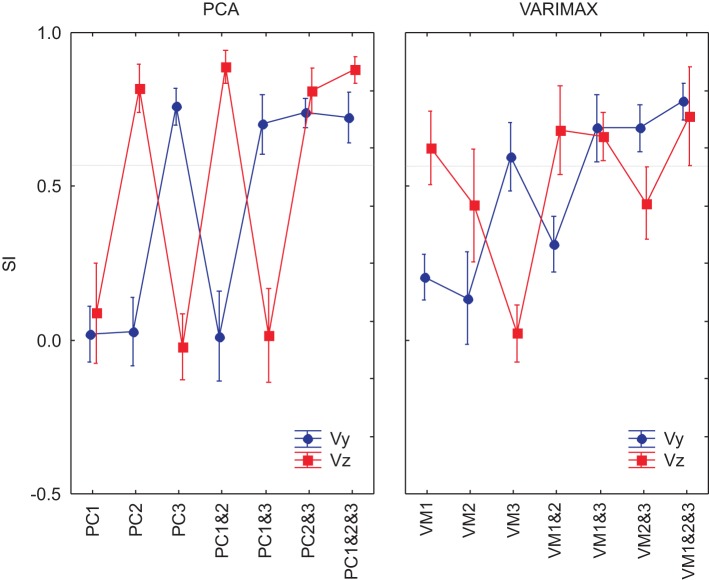
**SIs for EMG composed by PCs and VMs for an 8-pattern DRNN trained**. In blue, the SIs for the vertical component and in red the SIs for the horizontal component of the index-finger velocity.

We used ANOVA to test statistically the ability of each PCA method (unrotated or varimax) to reproduce the velocity profiles of the actual movements. We compared the similarity indexes between the 8 measured movement patterns for the reference subject with the simulated movements from the 7 different DRNN reconstructions from the corresponding EMG signals from the principal component (PC1, PC2, PC3, PC1&2, PC1&3, PC2&3, PC1&2&3) and varimax (VM1, VM2, VM3, VM1&2, VM1&3, VM2&3, VM1&2&3). This resulted in an ANOVA with two repeated-measures factors (EMG components X velocity component). Figure 8 illustrates the mean and SD values for SIs obtained for 8 measured figure-eight movements. In this figure we have drawn a “threshold” line that separate the SI values that present a significant difference from the best SIs values obtained with unrotated PCA for EMG composed with PC1&2. One observes that PC3 and VM3 were interpreted similarly as acting on the vertical component of the movement whereas VM1 acted more like PC2, each having a functional link with Vz. VM2 seems to have a more complex combination of information concerning both velocity components. When looking at the DRNN interpretation by combining PCs 2-by-2, one observes that PC2&3 and VM1&3 are interpreted similarly by DRNN as having the same level of action on Vy and Vz (Scheffe's *post-hoc p* < 0.99). The same situation is true for unrotated and varimax PC1&2. But the pattern of SIs for VM1&3 did not bear any resemblance to the patterns achieved with the principal components, since it reproduced better Vy than Vz. This observation, added to the fact that the mean SI for VM1&2 was not different from the one obtained for PC1&3, leads us to conclude that VM2 corresponded to a synergy that acts partly on horizontal and partly on vertical velocity component, without the clear demarcation between components that is found for unrotated principal components.

**Figure 9:**
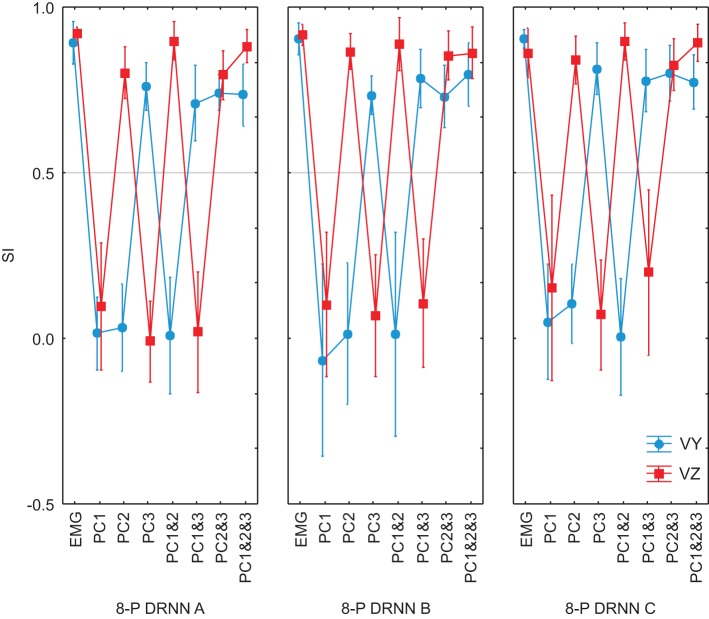
**SIs for EMG composed by PCs for 3 different instances of 8-pattern DRNNs trained from a random initial state**. Each graph represent the SIs for the simulations with the real EMG and the EMG composed by means of PC1, PC2, PC3, PC1&2, PC1&3, PC2&3, PC1&2&3. In black: the SIs for the vertical component and in gray the SIs for the horizontal component of the index-finger velocity.

We went further to ask whether the differences between the principal component and varimax decompositions in terms of the mapping of EMG components to hand velocity could be explained simply by differences in the particular instances of the trained DRNN, or whether the pattern is repeatable to any successfully trained instance of the DRNN. In Figure 9 we compared the behavior of 3 different instances of the DRNNs, all of which were trained with the same 8 movement trials from the reference subject, but each of which converged starting from a different random set of initial weights and time constants. Globally, the observations made for the single DRNN above were valid for all three instances of the DRNN. PC2 was associated with Vz while PC3 was associated with Vy. A Three-Way ANOVA, with PC combination, velocity component and DRNN instance showed no main effect of DRNN instance [*F*_(14, 84)_ = 1.4213, *p* = 0.16], nor any cross effects be DRNN instance and either of the other two independent factors.

#### DRNN generalization between subjects

**Figure 10:**
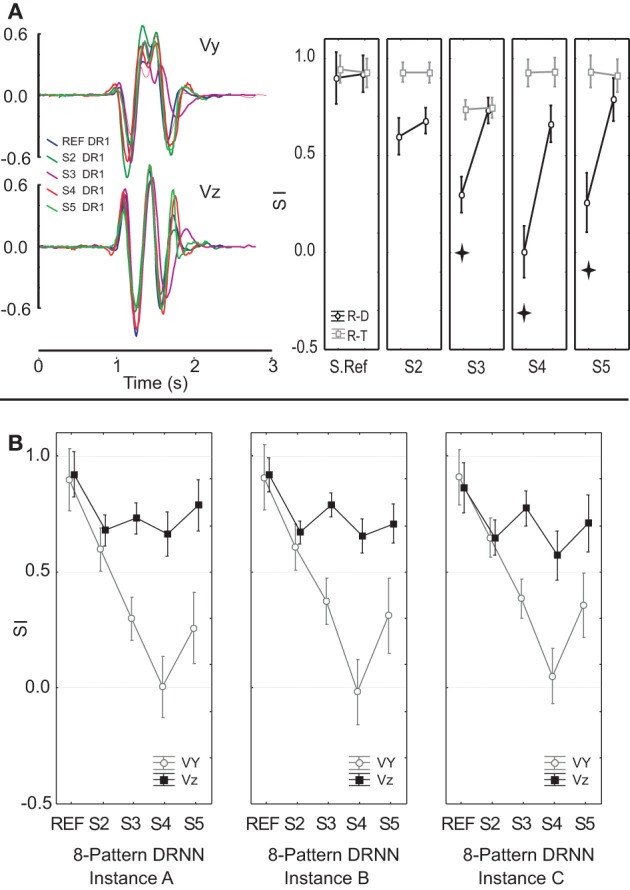
**(A)** An example of velocity profiles for a single movement pattern performed by 5 subjects. Similarity indexes for movements reconstructed from EMGs collected from each of 5 different subjects by an 8-pattern DRNN trained on data from the reference subject only. **(B)** Similarity indices for movements reconstructed by three different instances of an 8-pattern DRNN trained on data from the reference subject. Each instance converged to the final solution from a different set of random initial weights and time constants. Stars show SIs that were significantly different (*p* < 0.005).

We then tested the ability of a DRNN trained on data from one subject to simulate movements based on EMG signals recorded from another. We fed EMG signals from five different subjects to the same 8-pattern DRNN trained on movements from both planes performed by a single subject. The set of five subjects included the one subject whose data were used to train the networks, hereafter referred to as the reference subject, and four other subjects who performed the experiment in the frontal plane. Note that as in the previous analysis, the EMGs used to simulate movements for the reference subjects were different from that subject's EMG recordings used to train the network. The results of this analysis are shown in Figure 10A, where we have overlaid the velocity traces of for each subject and we have plotted the similarity indexes for the comparison of the actual movements and the movements predicted by the DRNN from the EMG (dark symbols). For comparison, we have plotted the SIs for the actual movements performed by each subject and the corresponding actual movements from the reference subject used to train the network (gray symbols). ANOVA showed that in general the DRNN predicted less well the velocity profiles of the other four subjects and that in all cases the SIs for Vy (Scheffe's *post-hoc*: *p* < 0.05) were lower than for Vz [cross-effect between subjects, velocities and real-trained movement vs. real-DRNN predictions *F*_(4, 49)_ = 15.156, *p* < 0.001]. This is in contrast to the between-subject comparisons of the actual movement profiles, which showed SIs that were greater than those observed for the DRNN-reconstructed movements and which did not differ between Vy and Vz.

We performed a two-factor ANOVA to test the ability of each DRNN to reproduce the measured hand velocities from the recorded EMGs. As before, *velocity component* (Vy and Vz) was treated as a repeated measure, to which was added the grouping factor *subject* to indicate which subject's EMG data was used to simulate each movement. This led to a 2 × 5 mixed-model ANOVA. The ANOVA also showed a significant difference in SIs between the five subjects [main effect of subject, *F*_(4, 49)_ = 28.439, *p* < 0.0001]. *Post-hoc* tests showed that the DRNN did a significantly better job, on average across both velocity components, of reproducing the trajectories for the reference subject (mean SI 0.91 ± 0.02) compared to all four other subjects (*p* < 0.00001). There was no overall difference (*p* > 0.3) for the simulations between three of the other subjects (mean SI was 0.64 ± 0.06, 0.52 ± 0.31 and 0.52 ± 0.37 for subject 2, 3, and 5 respectively). But subject 4 presented significantly lower SIs (*p* < 0.05) than any of the other subjects (mean SI 0.33 ± 0.46). There was, however, a significant cross effect between the subject and velocity-component factors [*F*_(4, 49)_ = 13.947, *p* < 0.0001]. Indeed, the main difference for the simulations between the reference subject and the other subjects was found for the vertical velocity component: Scheffe's *post-hoc* analysis showed a significant difference (*p* < 0.0001) for Vy between the reference subject (mean SI 0.9 ± 0.07) and three of the other four subjects (mean SI 0.3 ± 0.25, 0.004 ± 0.05, and 0.26 ± 0.19 for subjects 3, 4, and 5 respectively) but not for subject 2 (*p* < 0.054) (mean SI 0.60 ± 0.2 for subjects 2). For the horizontal component, Scheffe's *post-hoc* analysis showed no differences between the reference subject and the four others (mean SI were 0.92 ± 0.02, 0.68 ± 0.18, 0.73 ± 0.14, 0.66 ± 0.12, and 0.79 ± 0.03 for subjects 1, 2, 3, 4, and 5 respectively).

We went further to test whether the patterns in the mapping of EMG components to hand velocity was specific to this particular instance of a trained DRNN, or whether the pattern was repeatable to any successfully trained instance of the DRNN. In Figure 10B we compared the behavior of three different DRNN instances, all of which were trained with the same eight movement trials from the reference subject, but each of which converged starting from a different random set of initial weights and time constants. We performed an ANOVA on the similarity indices with *DRNN instance* (dual-plane A, dual-plane B, and dual-plane C) and *velocity component* (Vy and Vz) as repeated measures. To this was added the grouping factor *subject* to indicate which subject's EMG data were used to simulate each movement. This led to a 3 × 2 × 5 mixed-model ANOVA. There was no significant effect between the three instances of 8-pattern DRNNs [*F*_(2, 98)_ = 0.31392, *p* = 0.73131] nor was there a significant cross-effect between instances of 8-pattern DRNN and the factor subject [*F*_(8, 98)_ = 1.6801, *p* = 0.11275]. There was, however, a significant cross effect between the velocity and subject factors [*F*_(4, 49)_ = 16.861, *p* < 0.00001]. As we have observed in the other analyses, the main difference for the simulations between the reference subject and the other subjects was found for the vertical velocity component: Scheffe's *post-hoc* analysis showed a significant difference (*p* < 0.05) between the reference subject and each of the other 4 subjects for Vy but not for Vz. We note, finally, that the three-way interaction between subject, velocity component and 3 instances 8-pattern DRNN was not significant [*F*_(8, 98)_ = 1.0999, *p* = 0.36998].

**Figure 11:**
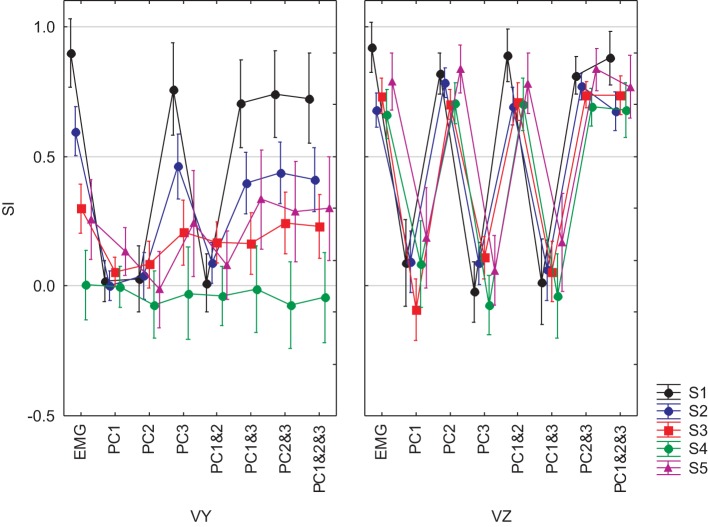
**SIs for EMGs composed from PCs for 5 subjects and for the 8-pattern DRNN**. The graphs in left and right columns show the SIs for vertical and horizontal velocity components, respectively. Each graph represent the SIs for the simulations with the real EMG and the EMG composed by means of PC1, PC2, PC3, PC1&2, PC1&3, PC2&3, PC1&2&3. Each of the 5 subjects is identified by a different color.

We completed our analysis by examining the ability of a DRNN trained on data from one subject to reproduce the hand trajectories of the other subjects on the basis of EMG signals reconstructed from different combinations of principal components. The similarity index was computed between each simulated movement and the corresponding actual movement and the SI's were subjected to a mixed-model ANOVA with *subject* as a grouping factor and *PC combination* (PC1, PC2, PC3, PC1&2, PC1&3, PC2&3, PC1&2&3, PC1–7) and *velocity component* (Vy, Vz) as repeated measures. This analysis showed that SIs depended on which subject's EMGs were fed to the DRNN [subject main effect: *F*_(4, 49)_ = 13.98, *p* < 0.0001], on which principal components were used to reconstruct the EMG (PC combination main effect: *F*_(7, 343)_ = 175.65, *p* < 0.0001] and on the velocity direction (velocity component main effect: *F*_(1, 49)_ = 91.757, *p* < 0.0001]. All interaction effects were highly significant (*p* < 0.0001). Figure 11 shows the overall results, from which one can make the following observations:

1. Reproduction of the horizontal velocity component depended on the presence of PC2 in the reconstructed EMG. For each subject, all SIs for Vz for reconstructions that included PC2 (PC2, PC1&2, PC2&3, PC1&2&3) were as good as the SI for Vz for the full EMG, while all SIs for Vy for EMGs that did not include PC2 (PC1, PC3, PC1&3) were equally bad.
2. Reproduction of the vertical velocity component depended on the presence of PC3 in the reconstructed EMG. For each subject, all SIs for Vy for reconstructions that included PC3 (PC3, PC1&3, PC2&3, PC1&2&3) were equally good as the SI for Vy for the full EMG and all SIs for Vz for EMGs that did not include PC3 (PC1, PC1&2) were equally bad.
3. The DRNN decoded the horizontal component of the hand velocity (Vz) just as well across all subjects as it did for the reference subject on whose data the network was trained. Compared to the reference subject, however, the DRNN did a much poorer job of reproducing the vertical component (Vy) for the 4 other subjects.

**Figure 12:**
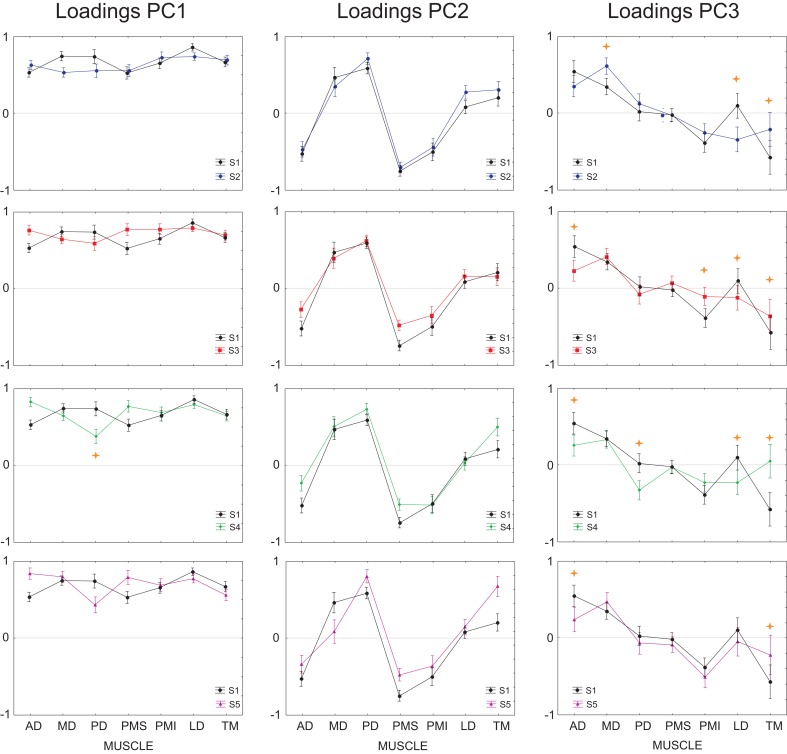
**Average loadings for subjects 2–5, compared to the average loading for the reference subject (subject 1)**. Individual muscle loadings that differed significantly between the reference subject and each of the other subjects are indicated with a 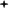
*p* < 0.001.

Finally, we tested whether the ability of the DRNN to predict movements for different subject could be related to differences between subjects in the loading vectors (synergies) identified by principal component analysis. We applied ANOVA to the loading vectors obtained in our companion study for each subject with *subject* as a grouping factor and *muscle* as a repeated measure. We limited this analysis to movements in the frontal plane. Figure 12 shows the average loadings for subjects 2–5, compared to the average loading for the reference subject (S1). Differences in individual muscle loadings that were significant (as measured by Scheffé's *post-hoc* test) are indicated with a ^*^. As shown in our companion article, the identified principal components were remarkably similar across subjects, with PC1 representing a global activation of all 7 muscles over the course of the movement, PC2 indicating a reciprocal relationship primarily between AD, PMS, and PMI on one side and MD and PD on the other, and PC3 showing a reciprocal relationship primarily between AD and MD on one side and PMI and TM on the other. Differences between the loadings of each subject and the loadings of the reference subject were restricted largely to PC3. This is consistent with the observation that (1) PC2 is linked to Vz and PC3 is linked to Vy and (2) that the DRNN predicted better Vz than Vy across subjects. There is nevertheless an indication of a tradeoff between the participation of TM, with this muscle sometimes participating primarily in PC2 and sometimes in PC3.

## Discussion

In this study we looked for modularity in patterns of drawing movements exemplified by the figure eights performed by our subjects. We used the training of a DRNN as a means to identify structure in the mapping from muscle activations to hand movements. While one might quibble over whether the DRNN has learned the dynamics of the arm, it is certain that the DRNN identified structure in the mapping of EMG signals to movements of the hand. Otherwise, it would not be able to predict movements from EMGs not included in the training set. But as we reported, the DRNN was not always able to predict movements from novel EMGs.

A failure of a trained DRNN to generalize to EMG inputs from outside the training set can arise either because the DRNN does not have enough degrees of freedom to learn the actual movement dynamics or because the training set does not contain enough contrasting information to reveal all the underlying structure. In our study, whenever the DRNN failed to generalize from a given, limited set of training movements, it always succeeded when presented with a broader set of examples during training. We concluded that the DRNN structure was sophisticated enough to capture the EMG-to-movement relationship, if presented with a rich enough training set. It is therefore interesting to contrast when the DRNN could and could not generalize from one dataset to another, as this provides an indicator as to what changes in conditions require modifications to the underlying movement-generating modules.

In this article we have also proposed a novel use of the DRNN to reveal properties of the modules that generate movement of the human arm. In the past we fed to the already trained DRNN modified versions of the individual EMG inputs that were scaled in amplitude (Cheron et al., 1996, 2003) or delayed in time (Cheron et al., 2007), in order to identify the action of individual muscles. In the current study we fed the trained DRNN with potential synergies that we identified through principal component analysis. This more global approach addressed the question of the neural organization of muscle activations from a modular point of view, in contrast to our preceding anatomical viewpoint concerning muscle mechanical actions.

### Spatial vs. temporal

As shown by de Rugy et al. (2013), variability in synergies could arise from specific behaviors or tasks to be accomplished. For the same motor program (a figure-eight) we should find in the EMG signals information corresponding to a general “figure-eight” motor program mixed with the information corresponding to the specific aspects of motor execution such as the movement's velocity, amplitude, joint configuration and biomechanical constraints. In this context, the cases where the DRNN failed to generalize can be summarized as follows: (1) the DRNN could not generalize from a single movement direction in a given workspace to the other three movements in the same workspace, (2) the DRNN could not generalize from movements performed in the four different directions in one workspace to the same four movement patterns in the other workspace, and (3) the DRNN could not generalize from one subject to another. One might hypothesize that the inability to generalize in any or all of these situations could be due to differences in the kinematics of the movements that were actually performed, i.e., the DRNN might fail to generalize if it has not been exposed to hand-velocity patterns in the training phase that are included in the test dataset. While this would be an interesting observation in itself, this was not the case in the study reported here. All the movements reported here corresponded to the realization of a figure eight. For the movements in different directions in the same plane, we have previously shown that the four movements were very similar in terms of spatial parameters and the temporal aspects of the trajectories (Cheron et al., 1999). Concerning the inter-workspace and inter-subject comparisons, the analysis of similarity indices between movements in the training and test datasets reported here (Figure 4) reject the hypothesis that the inability of the DRNN to generalize across these conditions can be attributed to differences in the movement patterns themselves. The differences detected by the DRNN between conditions must necessarily represent contrasts in the mapping between EMG and the movement in each situation.

Consider, then, the inability of the DRNN to generalize between movement directions within the same workspace. The spatial aspects of each of the four movements were the same, with the hand following the same spatial form (the figure eight), and the arm was fully outstretched in all cases. The biomechanical aspects of the different movements were therefore essentially constant, at least in terms of the moment arms and preferred directions for the muscles involved. The velocities and accelerations of the hand were also similar across the four different movement directions, although they were not presented in the same order within each of the different movements. If the mapping from movement to EMG required to generate that movement consists of a simple mapping of instantaneous position, velocity or acceleration to muscle activations, the DRNN should have been able to capture that simple, time-invariant relationship from any one of the four movements in the same plane. The fact that the DRNN was unable to generalize from one movement direction to another suggests, therefore, that the modules underlying movement generation must take into account the temporal aspects of muscle activation patterns (Ivanenko et al., 2004; d'Avella et al., 2008; Delis et al., 2014). As shown in our companion article concerning the synergy analyses of figure-eights, factor loadings of the first three PCs did not show any systematic differences with respect to different initial direction of movements, but the temporal components for PC2 and PC3 were modulated according to horizontal and vertical movement components, respectively.

The fact that the DRNN trained with 4 figure-eights realized only in the frontal workspace, was unable to generalize for the sagittal workspace (and vice-versa) could indicate that it was able to detect the biomechanical differences between the two workspaces and the related retuning of the modular commands (Hogan, 1985; Buneo et al., 1994; Cheung et al., 2005; Kamper et al., 2006; d'Avella et al., 2008). Similarly, even though the DRNN was able to generalize across all movements for the reference subject, if trained with all eight movement patterns, it was not able to predict movement patterns from EMGs taken from other subjects. In this context we note that across our 5 subjects the DRNN consistently associated the second principal component with the horizontal component of the finger velocity; and that the analyses of the loadings showed that for all the subjects this second PC revealed the same muscular synergy: a reciprocal activation of muscles according to their line of action in the horizontal plane. In contrast, the DRNN did not correctly predict movements from EMG generated with the third PC for anyone but the reference subject, and the loadings analyses showed significant differences for grouping muscles between them. Studies concerning kinematic and muscular synergies have already proposed that the lower PCs may be responsible for the general aspects of the movement and present less inter-individual variability whereas higher PCs would be responsible for more subtler aspects and by consequence it present more inter-individual variability (Santello et al., 1998; Torres-Oviedo and Ting, 2007; Frère and Hug, 2012). In the case of our vertical figure-eight the invariant aspect necessary to accomplish the trajectory would be expressed by the activation of PC1 and PC2 whereas the personal “signature” would be the consequence of the activation of PC3.

Exactly the same invariant identification was observed for the reference subject when we crossed the EMG/DRNN pairing. A 4-pattern DRNN trained on movements from one plane was able to reproduce the horizontal component of the finger velocity for EMG from movements in the other plane, but was unable to reproduce the vertical component. Factor loadings analyses showed that PC2 maintained the same muscular synergy across planes whereas the third PC loadings analyses showed significant differences for grouping muscles between planes. One can therefore conclude that the DRNN was able to detect directional, biomechanical and subject-dependencies in the mapping from EMG to movement (Muceli et al., 2010; Torres-Oviedo and Ting, 2010; Frère and Hug, 2012; Kristiansen et al., 2013). Indeed it associated directional dependencies to temporal tuning and biomechanical and subject dependencies to spatial tuning of the third PC.

### Horizontal vs. vertical

An interesting question emerges from the observation that the DRNN was much more able to reproduce the horizontal component of the hand's velocity than the vertical component. A 4-pattern DRNN trained on movements from one plane was able to reproduce the horizontal component of the finger velocity for EMG from movements in the other plane, but was unable to reproduce the vertical component. Similarly, an 8-pattern DRNN trained on data from one subject was able to reproduce, in most cases, the horizontal component but not the vertical component of movements produced by the other subjects. This observation indicates that the trained DRNN was able to identify an invariant aspect corresponding to the figure-eight, i.e., the control of the horizontal velocity.

Across all the movement conditions the only aspect that remained invariant was the fact that all the movements corresponded to the realization of a figure-eight. Across all the generalizations the only relationship that remained invariant was the identification of the synergy extracted by PC2 with the finger horizontal velocity component. This synergy corresponded to a reciprocal command that groups the shoulder muscles with respect to their horizontal preferred action direction. In a previous work (Bengoetxea et al., 2010) concerning the temporal activation pattern for a figure-eight, cross-correlation analyses showed that the invariant aspect across shoulder position and subjects was the emergence of two groups of muscles acting in a reciprocal mode in relation with the horizontal direction. This invariant synergy suggests the existence of an underlying oscillator module (Hogan and Sternad, 2012), acting in the horizontal direction, and the DRNN seems to have identified this module.

The analyses of the loading corresponding to PC3 across subjects indicates that the muscular synergies associated with the vertical component of the figure-eight were more variable, compared to the synergies defined by PC2 (see companion paper). We can offer several possible explanations of this observation. The first is purely methodological. The third principal component is by definition the one that explains the least amount of variance in the input signals, compared to the first and second. This means that the signal associated with this purported synergy would be smaller and thus more sensitive to noise. But that would seem unlikely to explain the enormous difference in the ability to predict the horizontal or vertical velocity components. The second is behavioral, as it could be that each subject has developed their own idiosyncratic synergies depending, for instance, on their professional activities or sports played. An alternative explanation may be found in the phasic/tonic aspects of a discrete figure-eight movement. For discrete reaching movement it has been shown that synergies are stable across subjects and shoulder positions (d'Avella et al., 2008). Modulations of synergies correspond to a cosine tuning for postural and tonic synergies and more complex pattern for phasic synergies. Tonic synergies are responsible for antigravity and postural control, whereas phasic synergies are responsible for overcoming inertia to accelerate and decelerate the arm (d'Avella et al., 2008). In our case the DRNN had to learn both tonic synergies, before and after movement, and phasic synergies during the movement. The fact that the DRNN was not able to generalize the vertical component of the movement across workspaces or subjects as well as their third PC could be due to the fact that the postural synergy and the phasic synergy for the vertical component of the movement were mixed.

Comparison of the principal component and varimax decompositions via the DRNN provides further fuel for our argument that muscle synergies for discrete-rhythmic movements are best captured by that identified by the principal components; i.e., one module controlling co-activation and two modules producing reciprocal activations, one in the horizontal and one in the vertical direction. When we fed the trained DRNN with EMG reconstructed by the first, second and the third PCs, we obtained a clear identification of each two spatial velocity components of the figure-eight movement. The reciprocal command extracted by PC2, where muscles were partitioned by their horizontal line of action, was clearly associated by the trained DRNN with the horizontal component of the finger velocity. Similarly, the reciprocal grouping by PC3, where muscles were partitioned by their vertical preferred direction, was associated by the trained DRNN with the vertical component of the finger velocity. The DRNN predicted little or no movement from an EMG signal constructed only from PC1, as would be expected from a co-activation module destined to tune the mechanical state of the system, rather to generate movement *per se*. The hand velocities predicted by the DRNN for the first three varimax components (VM1, VM2, VM3) were not nearly so well demarcated, with each producing a combination of vertical and horizontal velocity.

Using the DRNN to interpret the physiological meaning of the muscle synergies that were previously identified through principal component analysis is therefore an interesting addition to the tools that may be used to study modularity in movement control. One might say that the DRNN has captured to some degree the physiological and mechanical relationship between the muscles and the motor output. Of course, use of the DRNN cannot replace a thorough biomechanical model of muscle, bones and joints if one wishes to fully understand the mapping from EMG to movement. But like principal component analysis and other forms of factor analysis, the analysis by DRNN can be useful to identify structure in the underlying relationship, with the added advantage of linking muscle activation to actual movement and with the possibility of identifying causal relationships resulting from neural connections as well as from biomechanical constraints. The DRNN could potentially be coupled with other exploratory techniques, such as more recent efforts to identify modularity in temporal as well as spatial domains (s'Avella et al., 2003; d'Avella and Bizzi, 2005; Delis et al., 2014). Indeed, the “memory” elements of the DRNN have the potential to identify dynamical constraints that determine not only which muscles to activate for a given movement, but also when.

## Conclusions

A comparison of a DRNN's ability to generalize between movement conditions combined with principal component analysis suggests that tuning of movement-generation modules for movement direction seems to be related primarily to the temporal aspects of the movement whereas tuning to take into account joint biomechanics and inter-subject difference seems to be spatial (in the sense of how activity is spread between muscles). Analysis of the network's interpretation of synergies identified by principal component analysis provides further insight into how movement-generating modules are defined. This tool may therefore be used to motivate future experiments on the question of how human motor behavior may be organized in a modular fashion.

### Conflict of interest statement

The Guest Associate Editor Dr. Ivanenko declares that, despite having collaborated with authors Dr. Bengoetxea, Dr. Cheron, Dr. Dan and Dr. Hoellinger, the review process was handled objectively. The authors declare that the research was conducted in the absence of any commercial or financial relationships that could be construed as a potential conflict of interest.

## References

[B1] Bengoetxea A. Dan B. Leurs F. Cebolla A. M. de Saedeleer C. Gillis P. (2010). Rhythmic muscular activation pattern for fast figure-eight movement. Clin. Neurophysiol. 121, 754–765 10.1016/j.clinph.2009.12.021 20075001

[B2] Bicho E. Erlhagen W. Louro L. e Silva E. C. (2011). Neuro-cognitive mechanisms of decision making in joint action: a human-robot interaction study. Hum. Mov. Sci. 30, 846–868 10.1016/j.humov.2010.08.012 21208673

[B3] Bongaardt R. (2001). How Bernstein conquered movement, in Classics in Movement Science, eds Latash M. L. Zatsiorsky V. M. (Champaign, IL: Human Kinetics), 59–84

[B4] Buneo C. A. Soechting J. F. Flanders M. (1994). Muscle activation patterns for reaching: the representation of distance and time. J. Neurophysiol. 71, 1546–1558 8035234 10.1152/jn.1994.71.4.1546

[B5] Buneo C. A. Soechting J. F. Flanders M. (1997). Postural dependence of muscle actions: implications for neural control. J. Neurosci. 17, 2128–2142 9045739 10.1523/JNEUROSCI.17-06-02128.1997 PMC6793747

[B6] Cheron G. Bouillot E. Dan B. Bengoetxea A. Draye J. P. Lacquaniti F. (2001). Development of a kinematic coordination pattern in toddler locomotion: planar covariation. Exp. Brain Res. 137, 455–466 10.1007/s002210000663 11355390

[B7] Cheron G. Cebolla A. Leurs F. Bengoetxea A. Dan B. (2006). Development and motor control: from the first step on, in Progress in Motor Control and Learning. IV Development and Aging, eds Latash M. L. Lestienne F. (New York, NY: Springer), 127–139 10.1007/0-387-28287-4_12

[B8] Cheron G. Cebolla A. M. Bengoetxea A. Leurs F. Dan B. (2007). Recognition of the physiological actions of the triphasic emg pattern by a dynamic recurrent neural network. Neurosci. Lett. 414, 192–196 10.1016/j.neulet.2006.12.019 17224236

[B9] Cheron G. Draye J. P. Bengoetxea A. Dan B. (1999). Kinematics invariance in multi-directional complex movements in free space: effect of changing initial direction. Clin. Neurophysiol. 110, 757–764 10.1016/S1388-2457(99)00012-7 10378749

[B10] Cheron G. Draye J. P. Bourgeios M. Libert G. (1996). A dynamic neural network identification of electromyography and arm trajectory relationship during complex movements. IEEE Trans. Biomed. Eng. 43, 552–558 10.1109/10.488803 8849468

[B11] Cheron G. Duvinage M. Castermans T. Leurs F. Cebolla A. Bengoetxea A. (2011). Toward an integrative dynamic recurrent neural network for sensorimotor coordination dynamics, in Recurrent Neural Networks for Temporal Data Processing (Rijeka: Hubert Cardot. InTech), 65–80

[B12] Cheron G. Leurs F. Bengoetxea A. Draye J. P. Destrée M. Dan B. (2003). A dynamic recurrent neural network for multiple muscles electromyographic mapping to elevation angles of the lower limb in human locomotion. J. Neurosci. Methods 129, 95–104 10.1016/S0165-0270(03)00167-5 14511813

[B13] Cheung V. C. d'Avella A. Tresch M. C. Bizzi E. (2005). Central and sensory contributions to the activation and organization of muscle synergies during natural motor behaviors. J. Neurosci. 25, 6419–6434 10.1523/JNEUROSCI.4904-04.2005 16000633 PMC6725265

[B14] Cheung V. C. Piron L. Agostini M. Silvoni S. Turolla A. Bizzi E. (2009). Stability of muscle synergies for voluntary actions after cortical stroke in humans. Proc. Natl. Acad. Sci. U.S.A. 106, 19563–19568 10.1073/pnas.0910114106 19880747 PMC2780765

[B15] Cheung V. C. Turolla A. Agostini M. Silvoni S. Bennis C. Kasi P. (2012). Muscle synergy patterns as physiological markers of motor cortical damage. Proc. Natl. Acad. Sci. U.S.A. 109, 14652–14656 10.1073/pnas.1212056109 22908288 PMC3437897

[B16] Clark D. J. Ting L. H. Zajac F. E. Neptune R. R. Kautz S. A. (2010). Merging of healthy motor modules predicts reduced locomotor performance and muscle coordination complexity post-stroke. J. Neurophysiol. 103, 844–857 10.1152/jn.00825.2009 20007501 PMC2822696

[B17] d'Avella A. Bizzi E. (2005). Shared and specific muscle synergies in natural motor behaviors. Proc. Natl. Acad. Sci. U.S.A. 102, 3076–3081 10.1073/pnas.0500199102 15708969 PMC549495

[B18] d'Avella A. Fernandez L. Portone A. Lacquaniti F. (2008). Modulation of phasic and tonic muscle synergies with reaching direction and speed. J. Neurophysiol. 100, 1433–1454 10.1152/jn.01377.2007 18596190

[B19] d'Avella A. Portone A. Fernandez L. Lacquaniti F. (2006). Control of fast-reaching movements by muscle synergy combinations. J. Neurosci. 26, 7791–7810 10.1523/JNEUROSCI.0830-06.2006 16870725 PMC6674215

[B20] d'Avella A. Saltiel P. Bizzi E. (2003). Combinations of muscle synergies in the construction of a natural motor behavior. Nat. Neurosci. 6, 300–308 10.1038/nn1010 12563264

[B21] Delis I. Panzeri S. Pozzo T. Berret B. (2014). A unifying model of concurrent spatial and temporal modularity in muscle activity. J. Neurophysiol. 111, 675–693 10.1152/jn.00245.2013 24089400

[B22] de Rugy A. Loeb G. E. Carroll T. J. (2013). Are muscle synergies useful for neural control? Front. Comput. Neurosci. 7:19 10.3389/fncom.2013.00019 23519326 PMC3604633

[B23] Dominici N. Ivanenko Y. P. Cappellini G. d'Avella A. Mondì V. Cicchese M. (2011). Locomotor primitives in newborn babies and their development. Science 334, 997–999 10.1126/science.1210617 22096202

[B24] Doya K. (1996). Temporal difference learning in continuous time and space, in Advances in Neural Information Processing Systems 8, eds Touretzky D. S. Mozer M. C. Hasselmo M. E. (Cambridge, MA: MIT Press), 1073–1079

[B25] Draye J. P. Cheron G. Libert G. Godaux E. (1997). Emergence of clusters in the hidden layer of a dynamic recurrent neural network. Biol. Cybern. 76, 365–374 10.1007/s004220050350 9237362

[B26] Draye J. S. Pavisic D. A. Cheron G. A. Libert G. A. (1996). Dynamic recurrent neural networks: a dynamical analysis. IEEE Trans. Syst. Man Cybern. B Cybern. 26, 692–706 10.1109/3477.537312 18263069

[B27] Ferrigno G. Pedotti A. (1985). ELITE: a digital dedicated hardware system for movement analysis via real-time TV signal processing. IEEE Trans. Bio-Med. Eng. 32, 943–950 10.1109/TBME.1985.325627 3905583

[B28] Flanders M. Pellegrini J. J. Geisler S. D. (1996). Basic features of phasic activation for reaching in vertical planes. Exp. Brain Res. 110, 67–79 8817258 10.1007/BF00241376

[B29] Flanders M. Pellegrini J. J. Soechting J. F. (1994). Spatial/temporal characteristics of a motor pattern for reaching. J. Neurophysiol. 71, 811–813 8176443 10.1152/jn.1994.71.2.811

[B30] Frère J. Hug F. (2012). Between-subject variability of muscle synergies during a complex motor skill. Front. Comp. Neurosci. 6:99 10.3389/fncom.2012.00099 23293599 PMC3531715

[B31] Gentner R. Classen J. (2006). Modular organization of finger movements by the human central nervous system. Neuron 52, 731–742 10.1016/j.neuron.2006.09.038 17114055

[B32] Hof A. L. Van den Berg J. (1981). EMG to force processing i: an electrical analogue of the hill muscle model. J. Biomech. 14, 747–758 10.1016/0021-9290(81)90031-2 7334035

[B33] Hoffman D. S. Strick P. L. (1999). Step-tracking movements of the wrist. IV. Muscle activity associated with movements in different directions. J. Neurophysiol. 81, 319–333 9914292 10.1152/jn.1999.81.1.319

[B34] Hogan N. (1985). The mechanics of multi-joint posture and movement control. Biol. Cybern. 52, 315–331 10.1007/BF00355754 4052499

[B35] Hogan N. Sternad D. (2012). Dynamic primitives of motor behavior. Biol. Cybern. 106, 727–739 10.1007/s00422-012-0527-1 23124919 PMC3735361

[B36] Ivanenko Y. P. Poppele R. E. Lacquaniti F. (2004). Five basic muscle activation patterns account for muscle activity during human locomotion. J. Physiol. 556(Pt 1), 267–282 10.1113/jphysiol.2003.057174 14724214 PMC1664897

[B37] Kamper D. G. Fischer H. C. Cruz E. G. Rymer W. Z. (2006). Weakness is the primary contributor to finger impairment in chronic stroke. Arch. Phys. Med. Rehabil. 87, 1262–1269 10.1016/j.apmr.2006.05.013 16935065

[B38] Klein Breteler M. D. Simura K. J. Flanders M. (2007). Timing of muscle activation in a hand movement sequence. Cereb. Cortex 17, 803–815 10.1093/cercor/bhk033 16699078

[B39] Kristiansen M. Madeleine P. Hansen E. A. Samani A. (2013). Inter-subject variability of muscle synergies during bench press in power lifters and untrained individuals. Scand. J. Med. Sci. Sports. [Epub ahead of print]. 10.1111/sms.12167 24372591

[B40] Kuan C. M. Hornik K. (1991). Convergence of learning algorithms with constant learning rates. IEEE Trans. Neural Netw. 2, 484–489 18282861 10.1109/72.134285

[B41] Laje R. Buonomano D. V. (2013). Robust timing and motor patterns by taming chaos in recurrent neural networks. Nat. Neurosci. 16, 925–933 10.1038/nn.3405 23708144 PMC3753043

[B42] Liu J. K. Buonomano D. V. (2009). Embedding multiple trajectories in simulated recurrent neural networks in a self-organizing manner. J. Neurosci. 29, 13172–13181 10.1523/JNEUROSCI.2358-09.2009 19846705 PMC6665184

[B43] Muceli S. Boye A. T. d'Avella A. Farina D. (2010). Identifying representative synergy matrices for describing muscular activation patterns during multidirectional reaching in the horizontal plane. J. Neurophysiol. 103, 1532–1542 10.1152/jn.00559.2009 20071634

[B44] Rathelot J. A. Strick P. L. (2006). Muscle representation in the macaque motor cortex: an anatomical perspective. Proc. Natl. Acad. Sci. U.S.A. 103, 8257–8262 10.1073/pnas.0602933103 16702556 PMC1461407

[B45] Saltiel P. Wyler-Duda K. d'Avella A. Tresch M. C. Bizzi E. (2001). Muscle synergies encoded within the spinal cord: evidence from focal intraspinal NMDA iontophoresis in the frog. J. Neurophysiol. 85, 605–619 11160497 10.1152/jn.2001.85.2.605

[B46] Santello M. Flanders M. Soechting J. F. (1998). Postural hand synergies for tool use. J. Neurosci. 18, 10105–10115 9822764 10.1523/JNEUROSCI.18-23-10105.1998 PMC6793309

[B47] Song R. Tong K. Y. (2005). Using recurrent artificial neural network model to estimate voluntary elbow torque in dynamic situations. Med. Biol. Eng. Comp. 43, 473–480 10.1007/BF02344728 16255429

[B48] Tani J. Nishimoto R. Namikawa J. Ito M. (2008). Codevelopmental learning between human and humanoid robot using a dynamic neural-network model. IEEE Trans. Syst. Man Cybern. B Cybern. 38, 43–59 10.1109/TSMCB.2007.907738 18270081

[B49] Torres-Oviedo G. Ting L. H. (2007). Muscle synergies characterizing human postural responses. J. Neurophysiol. 98, 2144–2156 10.1152/jn.01360.2006 17652413

[B50] Torres-Oviedo G. Ting L. H. (2010). Subject-specific muscle synergies in human balance control are consistent across different biomechanical contexts. J. Neurophysiol. 103, 3084–3098 10.1152/jn.00960.2009 20393070 PMC2888239

[B51] Weiss E. J. Flanders M. (2004). Muscular and postural synergies of the human hand. J. Neurophysiol. 92, 523–535 10.1152/jn.01265.2003 14973321

[B52] Yi Z. Lv J. C. Zhang L. (2006). Output convergence analysis for a class of delayed recurrent neural networks with time-varying inputs. IEEE Trans. Syst. Man Cybern. B Cybern. 36, 87–95 10.1109/TSMCB.2005.854500 16468568

